# A Cytohistologic Correlation of Mucoepidermoid Carcinoma: Emphasizing the Rare Oncocytic Variant

**DOI:** 10.4061/2011/135796

**Published:** 2011-03-15

**Authors:** Timothy V. Wade, Virginia A. LiVolsi, Kathleen T. Montone, Zubair W. Baloch

**Affiliations:** Department of Pathology & Laboratory Medicine, University of Pennsylvania Medical Center, 6 Founders Pavilion, 3400 Spruce Street, Philadelphia, PA 19104, USA

## Abstract

It is well-known that the morphological variability of mucoepidermoid carcinoma (MEC) of the salivary glands may lead to interpretative difficulties on fine-needle aspiration (FNA) diagnosis. In this study we identify morphologic features that may be useful in the FNA diagnosis of MEC. The cohort included 23 cases of MEC; cytology and histology slides were reviewed and assessed for % cystic component, extracellular mucin, mucous and intermediate cells, oncocytes, cells with foamy/clear cytoplasm, keratinized cells and lymphocytes. On FNA 12/23 (52%) cases were diagnosed as consistent with or suggestive of MEC; 6/23 (26%) as salivary gland neoplasm and 5/23 (22%) as no tumor seen. The cystic component was ≥50% in 18/23 (78%) and <50% in 5 cases. The features prevalent in FNA and histology were: mucous cells (96% and 91%), extracellular mucin (91% both), intermediate cells (100 and 83%), lymphocytes (96 and 78%) and cells with foamy/clear cytoplasm (74% both). Oncocytes were seen in 43 and 22% and keratinized cells in 48 and 13% cases. Cases with oncocytes and lymphocytes were interpreted as favor Warthin's tumor on FNA. Presence of mucous cells, cells with foamy/clear cytoplasm, intermediate cells and lymphocytes in a mucinous background are diagnostic indicators of MEC; presence of oncocytes should not refrain from diagnosing MEC in FNA specimens.

## 1. Background

Fine needle aspiration (FNA) of salivary gland lesions is a valuable tool to pre-operatively diagnose/assess lesional tissue, determine the need for surgical intervention and assist in planning the appropriate surgical approach prior to resection. This technique is safe and effective with some studies demonstrating overall sensitivity, specificity and accuracy of 92%, 100%, and 98%, respectively [1–6]; however, the employment of FNA to diagnose mass lesions of the salivary glands remains controversial [1, 6]. The proponents believe that it can provide accurate diagnosis in many common tumors such as pleomorphic adenoma, distinguish benign from malignant lesions and prevent surgical intervention in cases with inflammatory lesions, lymphoma and certain metastatic tumors. The opponents of the use of FNA in salivary gland lesions believe that this procedure carries a high false negative rate and may fail to diagnose specific type of tumor. Many studies have shown that the latter is most likely due to the inherent morphologic variability that is, overlapping architectural patterns and nuclear cytology seen within these tumors, which can lead to interpretative difficulties on cytologic examination [1, 2, 7–10].

Mucoepidermoid carcinoma (MEC) is the most common malignant neoplasm of the salivary gland origin and accounts for 5% to 10% of all salivary gland neoplasms [11]. The majority of MEC occur in the parotid gland resulting in accessibility to biopsy by FNA; however, at times the diagnosis of MEC (mainly low-grade tumors by FNA) can be difficult due to overlapping cytomorphology with benign lesions [2, 3, 7, 8, 12, 13]. Therefore, given the common occurrence and heterogeneity of MEC, proper sampling and awareness of its morphologic complexity is critical to an accurate diagnosis. 

The difficulty in the cytologic diagnosis of MEC is related, in part, to the histologic grade of this tumor [7, 12]. High grade neoplasms are more easily recognizable as malignant and, therefore, more likely to receive the appropriate preoperative management [11]. By contrast, low-grade neoplasms are less easily recognizable as malignant and, therefore, under-diagnosis could result in treatment delays or inappropriate pre-operative management [14]. Numerous of grading schemes have been devised to differentiate between low, intermediate, and high-grade MEC [11, 14]. A scheme, proposed by Brandwein et al., assigned a numerical score to specific histologic features and adding these scores to determine the histologic grade. The accumulation of malignant features such as (tumor-type necrosis, nuclear pleomorphism, and high mitotic activity) results in a higher score [11]. Given the consequences of under-diagnosing MEC (such as treatment delays or inappropriate surgical approach), and the challenges of diagnosing MEC by cytology, in this study we attempt to identify the morphologic features that may be most useful in the FNA diagnosis of MEC, particularly of low-grade neoplasms.

## 2. Materials and Methods

In this retrospective study, 23 cases of MEC with preoperative FNA, were evaluated. The patient's ranged in age from 18 to 79 years and received surgical care at the Hospital of the University of Pennsylvania between 1995 and 2008. Cytology and histology slides and clinicopathologic features were reviewed in each case. The cases were assessed for the following features: % cystic component, nuclear atypia, necrosis, extracellular mucin, mucus cells, intermediate cells, oncocytes, and cells with foamy/clear cytoplasm, keratinized cells and lymphocytes. A histologic grade of low, intermediate or high was assessed in each case. The morphologic features noted by cytologic examination were compared to the original cytologic diagnosis in an effort to assess which features were the most consistent/reproducible in providing an accurate cytologic diagnosis and which histomorphologic features were associated with under-diagnosis of MEC by cytologic examination.

## 3. Results

In this study, 22/23 (96%) of, MECs arose in the parotid gland (average size 1.9 cm) and one (4%) from a minor salivary gland in the tongue. On FNA, 7/23 (30%) cases were diagnosed as consistent with, 5/23 (22%) as suggestive of MEC; 6/23 (26%) as salivary gland neoplasm and 5/23 (22%) as no tumor seen. In the six cases diagnosed as salivary gland neoplasm on FNA, two were diagnosed as favor acinic cell carcinoma (2/6), two were diagnosed as favor Warthin tumor (2/6), one was diagnosed as neoplasm with squamous differentiation (1/6) and another was diagnosed as favor benign mixed tumor versus mucoepidermoid carcinoma or adenoid cystic carcinoma (1/6). On histologic examination, the tumor grade was low in 13/23 (56%), intermediate 9/23 (39%) and high in 1/23 (4%) cases; neural invasion was seen in 4/23 (17%) and lymph node metastasis in 1/23 (4%) cases. The cystic component was ≥50% in 18/23 (78%) cases (see Tables 1 and 3). The morphologic features prevalent in both histology and FNA specimens included: mucus cells (96 and 91%), presence of extracellular mucin (91% both), intermediate cells (100 and 83%), lymphocytes (96 and 78%), and cells with foamy/clear cytoplasm (73% both). Oncocytic cells were seen in 43 and 22% and keratinized cells in 48 and 13% cases (see Table 2). 

An intraoperative frozen section was performed in 9/23 (39%) cases. The frozen section diagnoses were: MEC 4 cases, low-grade carcinoma 1, adenocarcinoma 1, consistent with Warthin tumor 1, cystic neoplasm 1, and no tumor seen 1 case. The average time interval between FNA and surgery was 5 weeks in 12 cases diagnosed as consistent with or suggestive of MEC. The average time interval was 4 weeks in 6 cases diagnosed as salivary gland neoplasm and 22 weeks in cases where FNA failed to identify tumor (see Tables 1 and 3).

## 4. Discussion

Mucoepidermoid carcinoma is the most common malignant salivary gland tumor. It is usually composed of varying amounts of epidermoid (squamoid) cells, intermediate cells, and mucocytes (often seen lining the microcysts). The combination of these cellular elements in varying proportions can lead to complex histologic patterns causing diagnostic challenges [11, 12]. The MEC are usually graded as low grade/welldifferentiated (tumor exhibiting greater than 50% of mucous elements), intermediate grade (10–50% of mucous elements and high grade (less than 10% of mucous elements). The histopathologic grading is usually used as the main prognostic indicator; however, some of the low-grade tumors can follow an aggressive clinical course [11, 14–18]. Furthermore, some experts believe that a tumor grading system of low and high grade is more reproducible as compared to the 3 category system [11]. 

Similar to histology, the diagnosis of low-grade MEC by FNA can be challenging due to spatial heterogeneity and multiple histologic components. Therefore, adequate sampling of various components within the tumor is essential to arrive at correct diagnosis [7, 12].

In our study, the morphologic features most prevalent in both the cytologic and histologic specimens of MEC were mucus cells (pseudo-goblet cells) and presence of extracellular mucin (both >90%). In addition, intermediate cells (100% in histology and 83% in cytology) and lymphocytes (96% in histology and 78% in cytology) were also commonly noted. The presence of oncocytic cells (43% in histology and 22% in cytology) and squamous/epidermoid cells (48% in histology and 13% in cytology) were less commonly seen. 

Oncocytic cells were seen 10/23 (43%) cases in histology and 5/23 (22%) of cytology cases. One case with oncocytic cells was interpreted on FNA as “salivary gland neoplasm favor Warthin tumor”, 2 as MEC, 1 as acinic cell carcinoma and 1 as suggestive of MEC. On re-review, all contained varying amounts of extracellular mucin, mucous cells and intermediate cells; pseudo-goblet cells/clear cells were seen in 3 cases. The case originally classified as Warthin tumors also contained an excess of lymphocytes. Oncocytic cells have been reported to occur in MEC; in addition, a rare variant of MEC known as oncocytic MEC has been described [19–22]. These tumors are composed exclusively of oncocytic cells arranged in nests and sheets in sclerotic stroma with variable number of chronic inflammatory cells [22]. The majority of the oncocytic MEC described in the literature lack or contain minimal squamous/epidermoid cells. On re-review we believe, based on the criteria described by Weinreb et.al, 5 of 10 cases represent MEC containing oncocytic cells as one the cellular components while 5 of 10 cases represent true oncocytic variant of MEC [22].

We believe that the most helpful features in differentiating MEC containing oncocytic cells from other salivary gland lesions in FNA specimens is the presence of extracellular mucin, mucous cells and pseudo-goblet/clear cells. 

Since a major difficulty in utilizing FNA to diagnose MEC is related to sampling, [7, 12] we believe it is most useful to identify various cellular and acellular components and formulate a differential diagnosis based on a few criteria including: nuclear atypia, metaplastic changes/cell type present (squamous, oncocytic, basal, or myoepithelial cells), presence or absence of lymphocytes, and presence of extracellular material (necrotic debris, chondromyxoid matrix or Mucin). Given the overlapping morphologic features of many salivary gland neoplasms, immunostains are rarely useful in differentiating the various salivary gland neoplasms [7, 23]. 

To add to these challenges, it has been shown that metaplastic/reparative changes can occur in benign salivary gland neoplasms due to physical trauma induced by FNA [24]. These changes include squamous metaplasia, infarction and necrosis, subepithelial stromal hyalinization, acute and chronic hemorrhage, inflammation with multinucleated giant cells, granulation tissue with subsequent fibrosis; cholesterol cleft formation, pseudoxanthomatous reaction, and microcystic degeneration. Thus, a repeat FNA of a salivary gland lesion containing above-mentioned reactive/reparative changes can pose an even greater challenge to the cytopathologist in the diagnosis of low-grade mucoepidermoid carcinoma; therefore, most clinicians will recommend surgery after an FNA diagnosis of salivary gland neoplasm [24]. 

Several studies have discussed the utility of intraoperative consultation in the surgical management of salivary gland lesions. Studies from our institution have shown that the diagnostic accuracy of FNA and frozen section are comparable for the interpretation of salivary gland neoplasms, and the accuracy of both is increased when used in conjunction [6]. In the current study, 12/23 (52%) cases were diagnosed as either consistent with or suggestive of MEC and 6/23 (26%) cases as salivary gland neoplasm. An intraoperative frozen section was performed in 9/23 (39%) cases, and of these, 8 (89%) cases were classified either as carcinoma or neoplasm (MEC 3, low-grade carcinoma 1, suggestive of MEC 1, adenocarcinoma 1, consistent with Warthin tumor 1, cystic neoplasm 1 and no tumor seen 1 case). Based on these data, the diagnostic accuracy of FNA is close to that of frozen section for the diagnosis of salivary gland neoplasms (78% (18/23) versus 89% (8/9)) and carcinomas (52% (12/23) versus 67% (6/9)). These findings support, as suggested by other authors, the combined use of intraoperative frozen section with FNA in the evaluation of salivary gland neoplasms [6].

## 5. Conclusions

We believe that the cytologic diagnosis of low-grade MEC remains challenging due to overlapping cytomorphologic features seen in other salivary gland lesions. Among the many cytologic features described, presence of extracellular mucin, mucous cells, and intermediate cells should raise the suspicion of MEC. In addition, oncocytic cells can occur in varying proportions in some cases of MEC and in the presence of above-mentioned features should not dissuade one from making or suggesting a diagnosis of this tumor in FNA specimens.

## Figures and Tables

**Figure 1:**
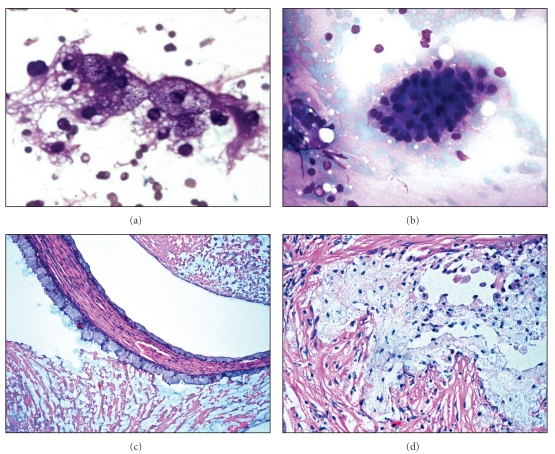
(a and b): High-power magnification of a Diff-Quik stained FNA specimen of low-grade mucoepidermoid carcinoma showing histiocyte-like (clear) cells with vacuolated cytoplasm (a) and fragments of glandular-type cells (b). (c and d) High-power magnification of H&E stained histologic section of a low-grade mucoepidermoid carcinoma showing glandular-type (mucous) cells lining a microcyst (c) and a cluster of histiocyte-like (clear) cells (d).

**Figure 2:**
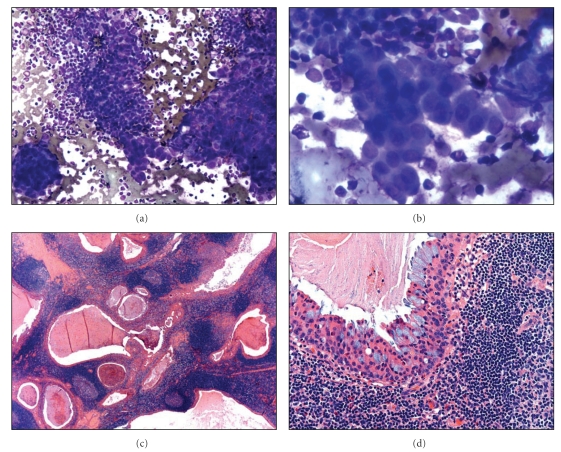
(a and b): Low-power magnification of a Diff-Quik stained FNA specimen showing oncocytic fragments present in a background lymphocytes (mistaken interpreted as Warthin's tumor) and high-power magnification of oncocytic fragments intermixed with lymphocytes. (c and d): Low-power magnification of H&E stained histologic section of a low-grade mucoepidermoid carcinoma showing a numerous cystic space lined by oncocytic epithelium in a background of reactive lymphoid follicles (c) and high-power magnification shows a microcyst lined by oncocytes and mucous cells (this case was misinterpreted as Warthins tumor on frozen section) (d).

**Table 1:** Clinicopathologic data on 23 patients with mucoepidermoid carcinoma.

FNA-Dx % cases	Gender (M : F)	Avg-Age (yrs)	Avg-Size (cm)	Location	Avg-time between FNA & resection
CW/SO MEC (12/23) 52%	8 : 4	49	1.6	11/12 parotid, 1 tongue	5 weeks
SGN (6/23) 26%	4 : 2	52	2.5	6/6 parotid	4 weeks
Neg or inflamm (5/23) 22%	1 : 4	50	1.6	5/5 parotid	22 weeks

Dx:Diagnosis, Avg:Average, CW:Consistent with, SO:Suggestive of, SGN:salivary gland neoplasm, Neg:Negative, Inflamm:Inflammatory.

**Table 2:** Key morphologic features observed in histology and FNA specimens.

Cell type observed	% in Histology	% in FNA
Mucous (pseudogoblet) cells	(22/23) 96%	(21/23) 91%
Lymphocytes	(22/23) 96%	(18/23) 78%
Clear cells	(17/23) 74%	(17/23) 74%
Intermediate cells	(23/23) 100%	(19/23) 83%
Keratinized squamous cells	(11/23) 48%	(3/23) 13%
Oncocytes	(10/23) 43%	(5/23) 22%

**Table 3:** Cytologic/Histologic diagnosis comparison.

FNA DX (23 cases)	FS DX (9/23 cases)	Histologic grade
C/W MEC (7/23)	MEC (3/5)	LGMEC (2/7)
	Adenocarcinoma (1/5)	IGMEC (4/7)
	Cystic Neop with papillary-features (1/5)	HGMEC (1/7)

S/O MEC (5/23)	SO MEC (1/2)	LGMEC (3/5)
	NTS (1/2)	IGMEC (2/5)

SGN: (6/23)	None performed	
Favor WT (2/6)		LGMEC (1/2)
		IGMEC (1/2)
Favor ACC(2/6)		LGMEC (2/2)
Favor BMT versus MEC or ADCC (1/6)		LGMEC (1/1)
Neop with squ-feat (1/6)		IGMEC (1/1)

Neg or inflamm (5/23)	LG-CA (1/2)	LGMEC (4/5)
	WT (1/2)	IGMEC (1/5)

Dx:Diagnosis, Avg:Average, CW:Consistent with, SO:Suggestive of, SGN:salivary gland neoplasm, Neg:Negative, Inflamm:Inflammatory.
